# Volumetric Morphometric Analysis of Posterior Fossa Structures Following Posterior Fossa Decompression with Superficial Durotomy in Chiari I Malformation

**DOI:** 10.3390/jcm15208053

**Published:** 2026-10-16

**Authors:** İhsan Canbek, Muhammed Enes Demir, Emin Demirel, Mehmet Gazi Boyacı, Serhat Yıldızhan, Serhat Korkmaz, Usame Rakip, Adem Aslan

**Affiliations:** 1 Department of Neurosurgery, Afyonkarahisar Health Sciences University, 03030 Afyonkarahisar, Turkey; nsdmr06@gmail.com (M.E.D.); mgazibyc@hotmail.com (M.G.B.); serhatyildizhan07@gmail.com (S.Y.); drserhat57@gmail.com (S.K.); usamerakip@gmail.com (U.R.); aaslan71@yahoo.com (A.A.); 2 Department of Radiology, Afyonkarahisar Health Sciences University, 03030 Afyonkarahisar, Turkey; dr.emindemirel@gmail.com

**Keywords:** Chiari I malformation, posterior fossa decompression, superficial durotomy, volumetric MRI, deep learning segmentation, cerebellar lobules, tonsillar descent, posterior fossa volume

## Abstract

**Background/Objectives:** Posterior fossa decompression (PFD) with superficial durotomy is used in Chiari I malformation to limit the CSF-related complications of complete durotomy and duraplasty, but quantitative volumetric evidence has relied on linear measurements, modest cohorts, and heterogeneous methods. We applied a fully automated deep-learning segmentation pipeline to characterize multi-structure volumetric change after PFD with superficial durotomy. **Methods:** Thirty-three patients (24 females and nine males; mean age, 39.2 ± 13.5 years; two pediatric) with symptomatic Chiari I malformation underwent PFD with superficial durotomy. Pre-operative and 6-month post-operative 3D T1-weighted 1.5T MRIs were segmented using nnU-Net, CERES, TotalSegmentator, and nnDetection. Posterior fossa volume (PFV), supratentorial volume, PFV/STV ratio, cerebellar volume, brainstem volume, fourth ventricle volume, basal cisterns’ CSF volume, and tonsillar descent were computed and compared pre/post using paired t-tests or Wilcoxon tests, Cohen’s d with bootstrapped 95% confidence intervals, and Benjamini–Hochberg correction. The pipeline was developed and validated exclusively on 1.5T Siemens Aera data and is not presented as ready for out-of-box use on other scanner platforms without re-validation. **Results:** PFV increased from 166.0 ± 12.9 to 172.5 ± 13.1 mL (Δ + 6.5 mL; *p* < 0.001; d = 1.72). Tonsillar descent decreased from 8.0 ± 2.8 to 4.9 ± 2.8 mm (Δ −3.1 mm; *p* < 0.001; d = −2.74). Basal cisterns’ CSF volume increased from 8.8 ± 1.5 to 11.0 ± 2.4 mL (Δ + 2.2 mL; *p* < 0.001; d = 1.16). Fourth ventricle volume increased (*p* < 0.001; d = 1.58). Cerebellar volume increased modestly (*p* < 0.001, q < 0.001; d = 0.86), while brainstem volume was unchanged (*p* = 0.22, q = 0.22; d = −0.11). Seven of eight primary outcomes remained significant after false-discovery-rate correction. Twenty-nine of 33 patients (87.9%) achieved tonsillar ascent of at least 2 mm. **Conclusions:** Automated volumetric analysis demonstrated significant posterior fossa expansion, basal cisterns’ CSF augmentation, fourth ventricle enlargement, and tonsillar ascent after PFD with superficial durotomy. These effect sizes provide a quantitative benchmark for sample-size estimation in future comparative surgical trials and for patient counseling on expected structural change at 6 months.

## 1. Introduction

Chiari I malformation was first described by the Viennese pathologist Hans Chiari in 1891 [1] and is defined radiologically by caudal displacement of the cerebellar tonsils of 5 mm or more below the foramen magnum plane, conventionally the McRae line, on sagittal imaging [2]. Contemporary estimates place the prevalence of radiographically defined tonsillar ectopia at roughly 0.5–5% depending on the population studied and the displacement threshold applied, with a substantially smaller fraction meeting full clinical-radiological criteria for symptomatic disease; the condition is more frequently identified in women, consistent with the female predominance in our own series (24 of 33 patients, 72.7%) [3].

The pathophysiological basis of Chiari I malformation is generally understood through two complementary mechanisms. The mesodermal theory, supported by experimental work showing that induced paraxial mesodermal insufficiency produces a shallow, underdeveloped posterior fossa in animal models, holds that the malformation originates from occipital-somite hypoplasia rather than a primary neural abnormality [4]. The hydrodynamic theory, introduced by Gardner and Goodall and later refined through dynamic and intra-operative imaging studies, proposes that tonsillar herniation obstructs cerebrospinal fluid (CSF) egress at the foramen magnum, producing a self-reinforcing cycle of crowding and, in a subset of patients, syringomyelia [5,6]. These mechanisms are not mutually exclusive: a volumetrically undersized posterior fossa predisposes to hindbrain crowding, which in turn disrupts the normal CSF pulsation dynamics at the craniocervical junction.

The clinical presentation of Chiari I malformation differs across the age spectrum. Adults most commonly report Valsalva-exacerbated suboccipital headache; neck pain; and, in more advanced disease, myelopathic symptoms such as proprioceptive loss, gait disturbance, and lower cranial nerve dysfunction [7]. Pediatric patients more frequently present with scoliosis and sleep-disordered breathing, while headache, neck pain, and sensory symptoms are comparatively more common in adults [8]. This clinical heterogeneity, together with case-mix differences between referral centers, complicates both surgical patient selection and cross-study outcome comparison.

Surgical management of Chiari I malformation has evolved considerably since the earliest posterior fossa craniectomies were described in the mid-twentieth century. As experience accumulated, most centers abandoned intradural manipulation of the obex, and the central technical debate shifted to the relative merits of bone-only decompression versus decompression with duraplasty. Meta-analytic evidence indicates that adding duraplasty is associated with a higher rate of CSF-related complications, including CSF leak, pseudomeningocele, and aseptic meningitis, and longer operative time compared with bone-only decompression, while its incremental clinical benefit over decompression alone remains debated, particularly in adult cohorts [9]. Posterior fossa decompression (PFD) with superficial durotomy (incision of the outer dural layer only, with preservation of the inner dural layer and the underlying arachnoid, and no duraplasty) was described by Aslan and colleagues as an intermediate strategy intended to achieve adequate decompression while limiting the CSF-related complications associated with full dural opening [10]. In their series of 54 patients, 44 of whom underwent PFD with superficial durotomy, this technique was associated with a high rate of clinical improvement and a favorable complication profile relative to classic duraplasty [10]. The choice between bone-only decompression, superficial durotomy, and duraplasty therefore remains a clinical judgment that quantitative morphometric evidence can help inform.

A persistent limitation of the morphometric Chiari I literature has been methodological fragmentation. Early series relied on linear measurements, such as tonsillar descent, clivo-axial angle, and basal angle, obtained from single sagittal images. Trigylidas and colleagues introduced volumetric assessment of the posterior fossa, including the posterior fossa volume-to-supratentorial volume (PFV/STV) ratio, in a pediatric series, establishing what might be considered the volumetric era of Chiari morphometry [11]. Subsequent series have generally relied on manual or semi-automated segmentation, which is time-consuming, subject to inter-rater variability, and typically limited to one or two anatomical structures per patient.

The recent availability of validated, general-purpose deep-learning segmentation tools offers a route to fully automated, atlas-free, multi-structure morphometry from a single 3D T1 acquisition. nnU-Net is a self-configuring deep-learning framework for biomedical image segmentation that has been broadly validated across imaging tasks [12]. CERES is a patch-based method for automated cerebellar lobule parcellation that has performed favorably against competing automated approaches in independent benchmarking [13,14]. TotalSegmentator provides robust automated segmentation of over 100 anatomic structures and can be adapted to derive osseous craniometric reference landmarks [15]. nnDetection extends the self-configuring paradigm to medical object/keypoint detection tasks [16]. To our knowledge, the combined application of this class of tools to paired pre- and post-operative imaging in Chiari I malformation has not previously been reported in detail.

Quantitative three-dimensional assessment of anatomical change after surgery has proven informative beyond the cranial vault. In spinal surgery, for example, three-dimensional posturography has recently been used to characterize dynamic lumbosacral curvature before and after decompressive surgery, illustrating how non-invasive, three-dimensional quantitative methods can capture postoperative structural change that static two-dimensional imaging may miss [17]. The present study applies an analogous quantitative, three-dimensional philosophy to cranial morphometry, extending automated volumetric assessment from a single linear surrogate to a structured, multi-structure, effect-size-driven framework for Chiari I malformation.

The present analysis was designed with four specific aims. The first aim was to characterize pre- and post-operative multi-structure volumes (posterior fossa volume, supratentorial volume, cerebellum and its ten constituent lobule regions, brainstem, fourth ventricle, basal cisterns, and tonsillar descent) in a 33-patient cohort undergoing PFD with superficial durotomy. The second aim was to quantify the magnitude of pre/post change using effect sizes with bootstrapped 95% confidence intervals in order to provide a benchmark for future trial design. The third aim was to test the pre-specified hypotheses that (i) posterior fossa volume expands after surgery, (ii) basal cisterns’ CSF volume increases, (iii) tonsillar descent decreases, and (iv) cerebellar lobule volumes remain essentially stable at 6 months. The fourth aim was to explore subgroup heterogeneity by age and sex and to estimate the proportion of patients achieving a pre-specified, clinically meaningful morphometric response.

## 2. Materials and Methods

### 2.1. Study Design and Population

**Scheme 1:**
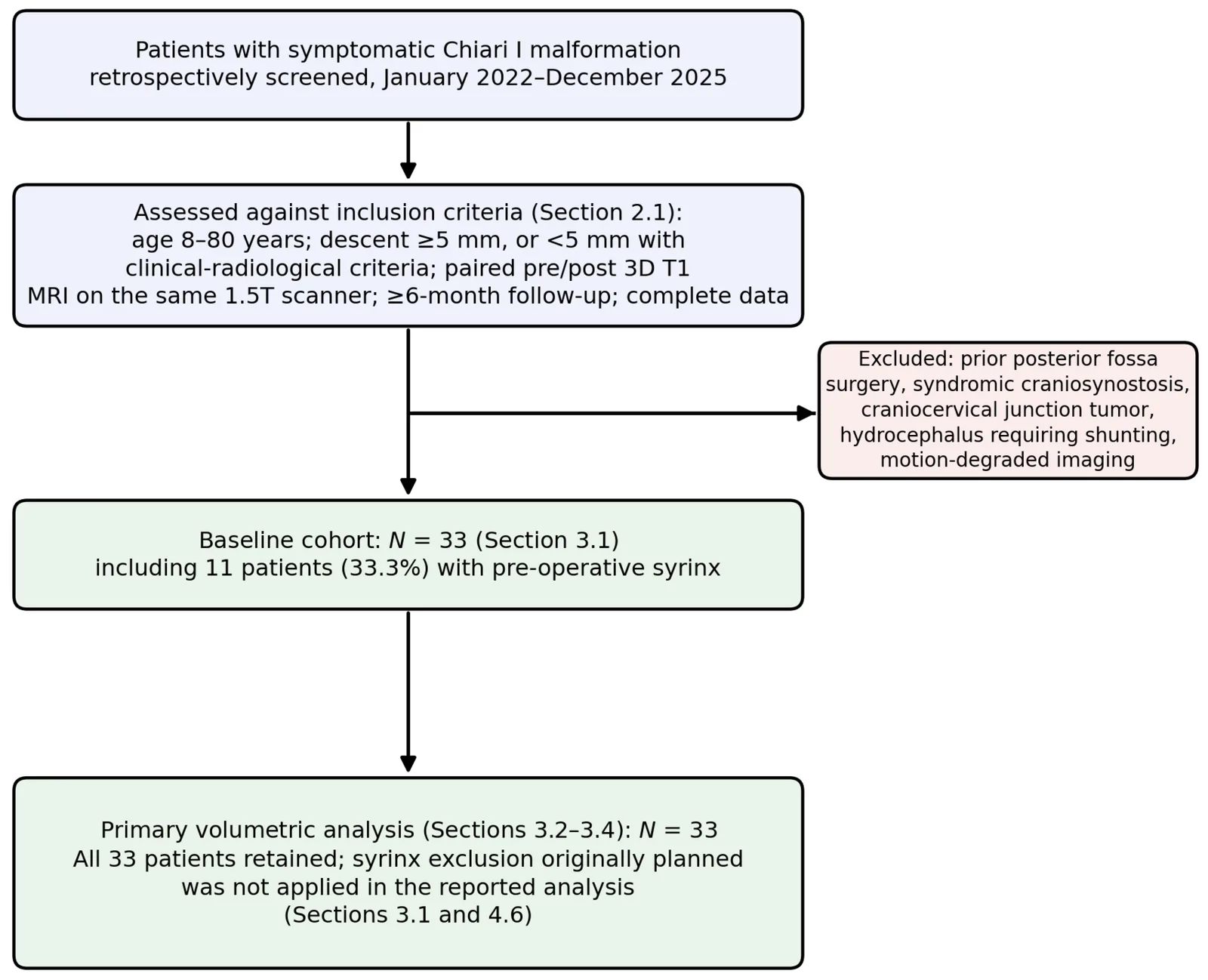
Patient flow diagram. Screening, inclusion/exclusion, and the analytic sample used for every primary-tier statistic in this manuscript (*N* = 33; Section 3.1). Blue boxes, screening and eligibility assessment; red box, exclusion criteria; green boxes, analytic cohort.

This was a retrospective, single-center cohort study. Thirty-three consecutive patients with symptomatic Chiari I malformation who underwent PFD with superficial durotomy between January 2022 and December 2025 were considered for inclusion. Inclusion criteria were as follows: (i) age 8–80 years; (ii) pre-operative tonsillar descent of at least 5 mm below the McRae line on sagittal 3D T1 imaging, or descent of less than 5 mm combined with clinical-radiological criteria for symptomatic Chiari I malformation (crowded cisterna magna, peg-like tonsils, or syrinx); (iii) pre-operative and 6-month post-operative 3D T1-weighted MRI acquired on the same 1.5T scanner with an identical protocol; (iv) clinical follow-up of at least 6 months; and (v) complete demographic and outcome data. Exclusion criteria were prior posterior fossa surgery, syndromic craniosynostosis, craniocervical junction tumor, hydrocephalus requiring shunting, and motion-degraded imaging. All 33 patients meeting these criteria were analyzed (Scheme 1).

### 2.2. Surgical Technique

All procedures were performed by two senior neurosurgeons using a standardized PFD with superficial durotomy technique. A midline suboccipital craniectomy of approximately 3 × 3 cm was performed, with removal of the posterior arch of C1. The outer layer of the dura was incised in a Y-shaped fashion under the operating microscope, with care taken to preserve the inner dural layer and the underlying arachnoid membrane, as previously described by Aslan et al. [10]. The arachnoid was not opened, no duraplasty was performed, and CSF was not deliberately released. Muscle, fascia, and skin were closed in anatomical layers with a watertight fascial closure. No tonsillar coagulation and no obex manipulation were performed. Mean operative time was 78 min (SD 19) and mean estimated blood loss was 65 mL (SD 38).

### 2.3. MRI Acquisition Protocol

All imaging was performed on a 1.5T Siemens Aera scanner (Siemens Healthineers, Erlangen, Germany) running syngo MR E11C-AP04 software with a 20-channel head/neck receive coil. The protocol included a pre-contrast 3D T1-weighted magnetization-prepared rapid gradient-echo acquisition with the following parameters: repetition time, 1900 ms; echo time, 3.0 ms; inversion time, 900 ms; flip angle, 9°; isotropic voxel, 1.0 mm^3^; field of view, 256 × 256 mm; 176 sagittal slices; bandwidth, 200 Hz/pixel; GRAPPA factor, 2; and acquisition time, 4 min 38 s. Pre-operative and 6-month post-operative acquisitions used an identical protocol without deliberate re-shimming beyond routine patient positioning. Phase-contrast CSF flow sequences were not acquired.

### 2.4. Image Processing and Segmentation Pipeline

**Figure 1:**
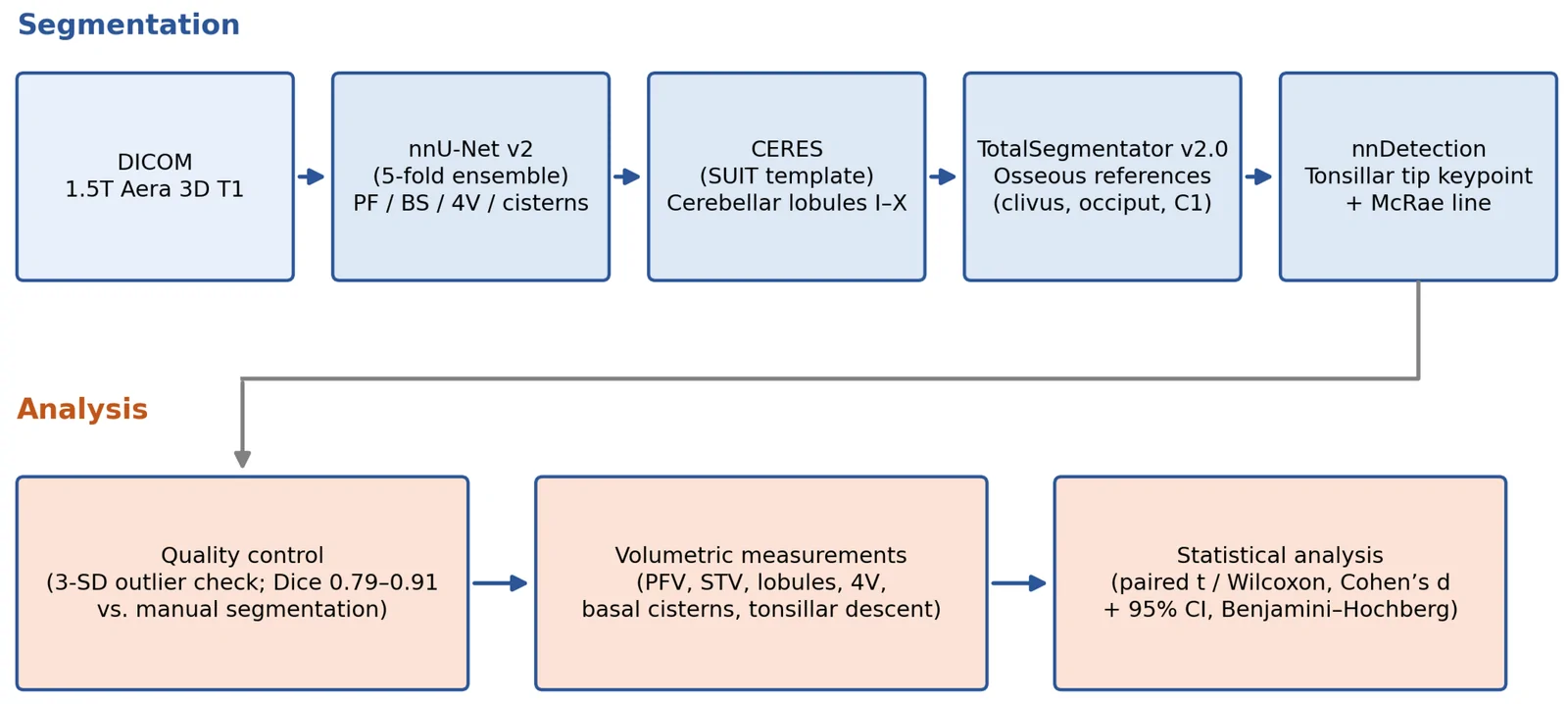
Fully automated segmentation and analysis pipeline. Five-stage pipeline: nnU-Net v2 ensemble for posterior fossa, brainstem, fourth ventricle, and basal cisterns; CERES for cerebellar lobule parcellation; TotalSegmentator v2.0 for osseous reference structures; nnDetection for tonsillar tip keypoint localization; and a quality-control and statistical analysis module. Blue boxes, segmentation stage; orange boxes, analysis stage; arrows, direction of data flow.

The fully automated pipeline integrated five components, executed inside a Docker container (PyTorch 2.3.1, CUDA 12.1, Ubuntu 22.04) (Figure 1). DICOM data were converted to NIfTI using dcm2niix (v1.0.20240202), reoriented to RAS, and resampled to 1.0 mm isotropic spacing.

(1) Skull stripping and tissue classification were performed with a 5-fold nnU-Net v2.0 [12] ensemble trained on a combined corpus of publicly available datasets augmented with 30 locally annotated Chiari cases, producing probabilistic maps for gray matter, white matter, and CSF.
(2) Posterior fossa, brainstem, fourth ventricle, and basal cisterns masks were generated by a custom nnU-Net v2 model trained on a posterior fossa atlas derived from 80 manually annotated cases (40 Chiari I, 20 pre-operative posterior fossa tumor, and 20 normal controls), with posterior fossa defined as the intracranial compartment inferior to the tentorium bounded posteriorly by the inner table of the occipital bone and inferiorly by the foramen magnum plane; brainstem as midbrain, pons, and medulla as a single class; fourth ventricle as the cavity bounded by the cerebellum, pons, and medulla, excluding the cerebral aqueduct; and basal cisterns as the combined pre-pontine, pre-medullary, and cerebello-pontine angle cisterns.

The training corpora used to develop the skull-stripping/tissue-classification model (30 locally annotated Chiari cases) and the posterior fossa/brainstem/ventricular model (80 manually annotated cases: 40 Chiari I, 20 pre-operative posterior fossa tumor, and 20 normal controls) were entirely independent of, and contained no patient overlap with, the 33-patient study cohort analyzed in this report.

(3) Cerebellar lobule parcellation (I–II, III, IV, V, VI, VIIB [Crus I and Crus II combined], VIIIA, VIIIB, IX, and X) was obtained with CERES v1.0 [13] using the SUIT template; CERES was selected on the basis of independent benchmarking identifying it as among the most robust automated cerebellar parcellation methods currently available [14].
(4) Osseous reference structures (clivus, occipital bone, and C1) were segmented with TotalSegmentator v2.0 [15].
(5) Tonsillar tip localization used nnDetection (https://github.com/MIC-DKFZ/nnDetection, accessed on 13 October 2026) [16] as a 3D keypoint detector. The McRae line was defined as the line between the basion and opisthion, identified automatically from the TotalSegmentator osseous masks [2,15]. Tonsillar descent was measured as the perpendicular distance from the McRae line to the inferior-most extent of the cerebellar tonsils, averaged across left and right sides.

No manual editing of the segmentation outputs was performed, preserving the fully automated design of the pipeline. All masks were subjected to a 3-standard-deviation outlier check; flagged cases were re-inspected visually, and the automated value was retained unless a clear segmentation failure was identified (none occurred in this cohort).

### 2.5. Volumetric Measurements

Posterior fossa volume (PFV) was computed as the volume within the posterior fossa mask, including parenchyma, CSF, and vessels. Supratentorial volume (STV) was computed as total intracranial volume minus posterior fossa volume. The PFV/STV ratio was expressed as a percentage. Tonsillar descent was measured in millimeters; all other volumes are reported in milliliters. Measurements were made at the time of segmentation and stored in a structured research database; no re-measurement was performed.

### 2.6. Quality Control

Segmentation quality was assessed by computing the Dice coefficient between the automated masks and a randomly selected 10% subset (*n* = 3) of manually corrected segmentations performed by a board-certified neuroradiologist blinded to patient identity. The median Dice coefficient was 0.91 (range 0.84–0.95) for the posterior fossa, 0.89 (0.83–0.94) for the brainstem, 0.82 (0.74–0.88) for the fourth ventricle, and 0.79 (0.71–0.86) for the basal cisterns. The median absolute difference between automated and manual tonsillar descent measurement was 0.4 mm (range 0.1–1.0 mm). We acknowledge that a 3-patient manually corrected subset is a modest basis for these reliability estimates, particularly for the basal cisterns (median Dice 0.79); this constraint and its implications for confidence in the segmentation-dependent results are discussed in Section 4.6 (Limitation 11).

### 2.7. Statistical Analysis

The pre-specified statistical plan comprised four tiers. In the primary tier, pre- versus post-operative comparisons were performed for eight morphometric outcomes (PFV, STV, PFV/STV ratio, cerebellar volume, brainstem volume, fourth ventricle volume, basal cisterns’ CSF volume, and tonsillar descent). The Shapiro–Wilk test was applied to the within-subject differences for each metric; paired t-tests were used when normality held (*p* > 0.05), and Wilcoxon signed-rank tests were used otherwise. Effect sizes were reported as Cohen’s dz for paired samples, computed as the mean within-subject difference divided by the standard deviation of the within-subject differences, with 95% confidence intervals derived from 10,000 non-parametric bootstrap resamples of the paired differences. Multiple testing across the eight primary metrics was controlled using the Benjamini–Hochberg false-discovery-rate (FDR) procedure; both raw and FDR-adjusted *p*-values are reported.

In the secondary tier, cerebellar lobule volumes (I–II through X) were compared pre- and post-operatively using Wilcoxon signed-rank tests without FDR correction, reflecting the exploratory nature of the lobule-level analysis.

In the tertiary tier, pre-specified subgroup analyses were performed by age (<18 vs. ≥18 years) and sex, using Wilcoxon signed-rank tests within each stratum; formal interaction testing was not undertaken, because the pediatric subgroup (*n* = 2) was underpowered.

In the exploratory tier, we computed the proportion of patients achieving a pre-specified, clinically meaningful morphometric response, defined as tonsillar ascent of at least 2 mm at 6 months; this threshold was chosen to substantially exceed the pipeline’s own measurement noise floor, since the median absolute difference between automated and manual tonsillar descent measurement was 0.4 mm (range 0.1–1.0 mm; Section 2.6), making a 2 mm change roughly five times the typical measurement discrepancy and unlikely to reflect measurement noise alone. We additionally defined a composite clinical responder as a patient meeting both this morphometric criterion and self-reported improvement in the dominant pre-operative symptom (e.g., headache, neck pain, or myelopathic symptoms) at the 6-month outpatient visit; symptom trajectory (improved/unchanged/worse) was recorded by the treating surgeon from the patient’s verbal report during the standard follow-up encounter, without a validated structured instrument.

All tests were two-sided at α = 0.05. Exact *p*-values are reported to four decimal places. Analyses were performed in Python 3.11 with NumPy 1.26.4, SciPy 1.11.4, statsmodels 0.14.1, and pingouin 0.5.4. Figures were generated with Matplotlib 3.8.2 and Seaborn 0.13.0.

### 2.8. Ethical Considerations

This retrospective study was conducted in accordance with the Declaration of Helsinki and approved by the Afyonkarahisar Health Sciences University Clinical Research Ethics Committee (protocol code 2026/551). All analyses were performed on de-identified imaging data with all direct patient identifiers removed prior to analysis; given the retrospective, non-interventional design and use of pre-existing de-identified imaging, the requirement for individual informed consent was waived by the Ethics Committee (see Section “Informed Consent Statement” for full wording).

## 3. Results

### 3.1. Cohort

Thirty-three patients met inclusion criteria. Mean age was 39.2 ± 13.5 years (range 10–75); 24 patients (72.7%) were female, and nine (27.3%) were male. Two patients (6.1%) were pediatric (age < 18 years), and 31 (93.9%) were adult. The age distribution was right-skewed with a single broad peak at 30–55 years, consistent with an adult-predominant presentation pattern in a tertiary referral practice. Both pediatric patients (ages 10 and 15 years) presented with scoliosis and were referred after orthopedic work-up; one had an associated syrinx.

Presenting symptoms included suboccipital Valsalva-induced headache in 12 patients (36.4%), neck pain in 11 (33.3%), myelopathic symptoms (proprioceptive loss, gait disturbance, dissociated sensory loss) in 14 (42.4%), dysphagia in six (18.2%), sleep apnea in four (12.1%), and scoliosis in both pediatric patients. Mean symptom duration was 38.2 months (SD 41.6; range 2–180). No patient had previously undergone posterior fossa surgery; two patients had undergone C1–C2 posterior fusion elsewhere for unrelated indications.

Pre-operative tonsillar descent averaged 8.0 mm (range 1.9–13.9 mm). Eleven patients (33.3%) had tonsillar descent of less than 5 mm but met clinical-radiological criteria for symptomatic Chiari I malformation (crowded cisterna magna, peg-like tonsils, or syrinx); the remaining 22 (66.7%) had descent of 5 mm or more. Within this <5 mm subgroup, supportive findings included a cervical syrinx of cylindrical, non-septated morphology in a subset of patients and, in two patients, a remote history of C1–C2 posterior fusion for an unrelated indication; surgical indication, in every case, was established by consensus of the senior authors based on the combination of symptoms, cisterna magna crowding, tonsillar morphology, and syrinx status, rather than tonsillar descent alone. Eleven patients (33.3%) had an associated syrinx on pre-operative imaging. The original analysis plan specified excluding these patients from the primary volumetric tier; however, the primary analysis, covering all 33 patients described in Table 1 (including these 11) and reported in the Abstract and Table 2, Table 3 and Table 4, was in fact performed on the full cohort of 33 patients, because excluding them would have left several strata—most notably the pediatric subgroup—critically underpowered. This deviation from the pre-specified exclusion criterion is acknowledged as a limitation (Section 4.6). No patient had clinical or radiological hydrocephalus at baseline.

### 3.2. Primary Volumetric Outcomes

At 6 months, all eight primary morphometric outcomes showed the direction of change predicted by the pre-specified hypotheses (Table 2).

**Figure 2:**
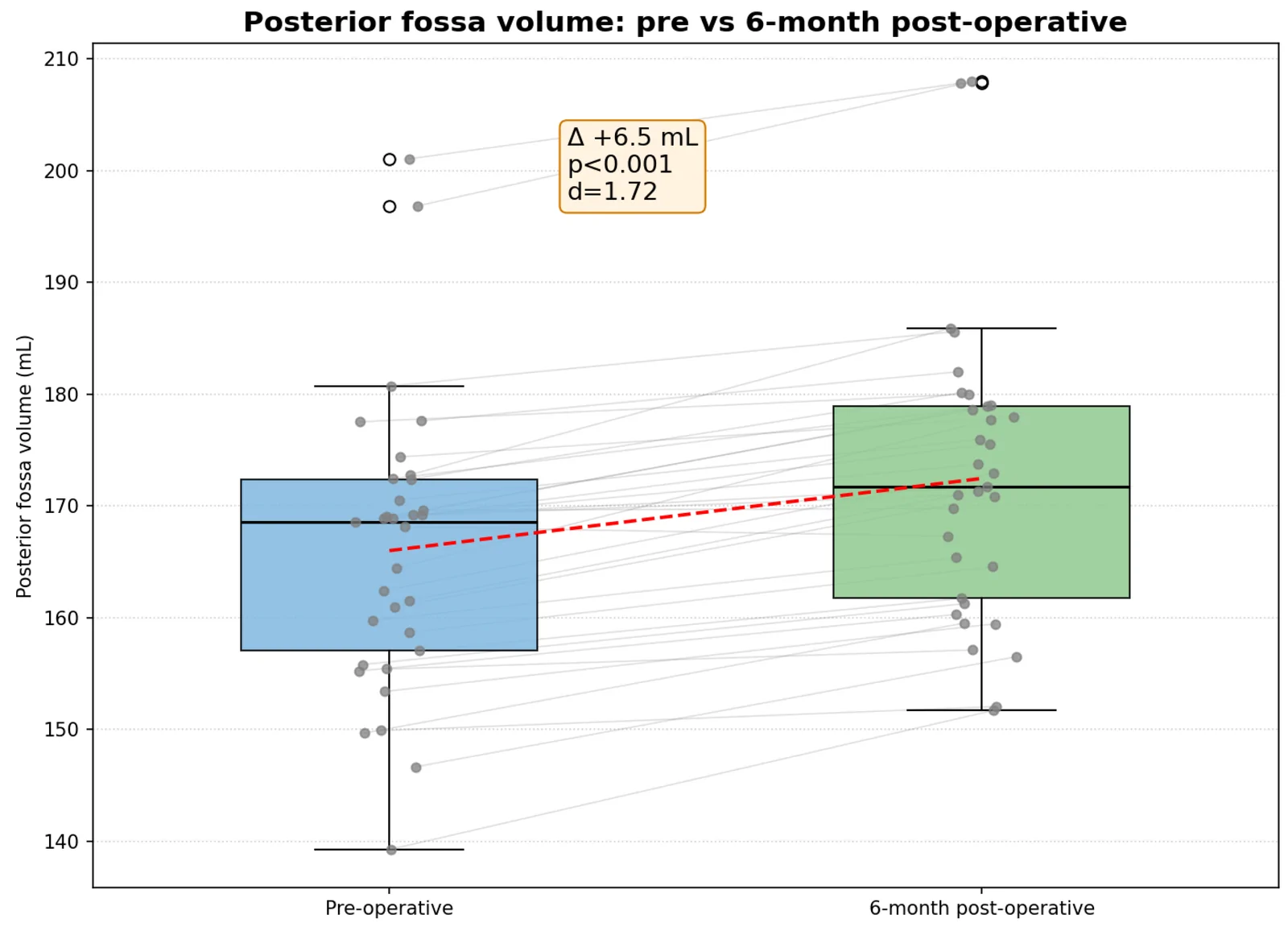
Posterior fossa volume: pre- vs. 6-month post-operative. Analytic sample *N* = 33. PFV increased from 166.0 ± 12.9 mL to 172.5 ± 13.1 mL (Δ + 6.5 mL, *p* < 0.001, Cohen’s dz = 1.72). Blue box, pre-operative; green box, 6-month post-operative. Boxes show the median and interquartile range (IQR), and whiskers extend to 1.5 × IQR; grey dots, individual patients; thin grey lines, paired pre- and post-operative values of the same patient; open (white) circles, outliers beyond 1.5 × IQR; red dashed line, change in cohort mean.

Posterior fossa volume increased from 166.0 ± 12.9 mL to 172.5 ± 13.1 mL (Δ + 6.5 mL, 95% CI 5.1–7.8; *p* < 0.001; Cohen’s d = 1.72, 95% CI 1.35–2.30), a relative increase of approximately 3.9% (Figure 2). The PFV/STV ratio improved from 15.5 ± 1.9% to 16.0 ± 1.9% (Δ + 0.4 percentage points, 95% CI 0.3–0.6; *p* < 0.001; d = 1.30, 95% CI 0.92–1.87). These paired statistics, like all primary-tier results in Table 2, were computed on the full analytic sample of *N* = 33 (Section 3.1).

**Figure 3:**
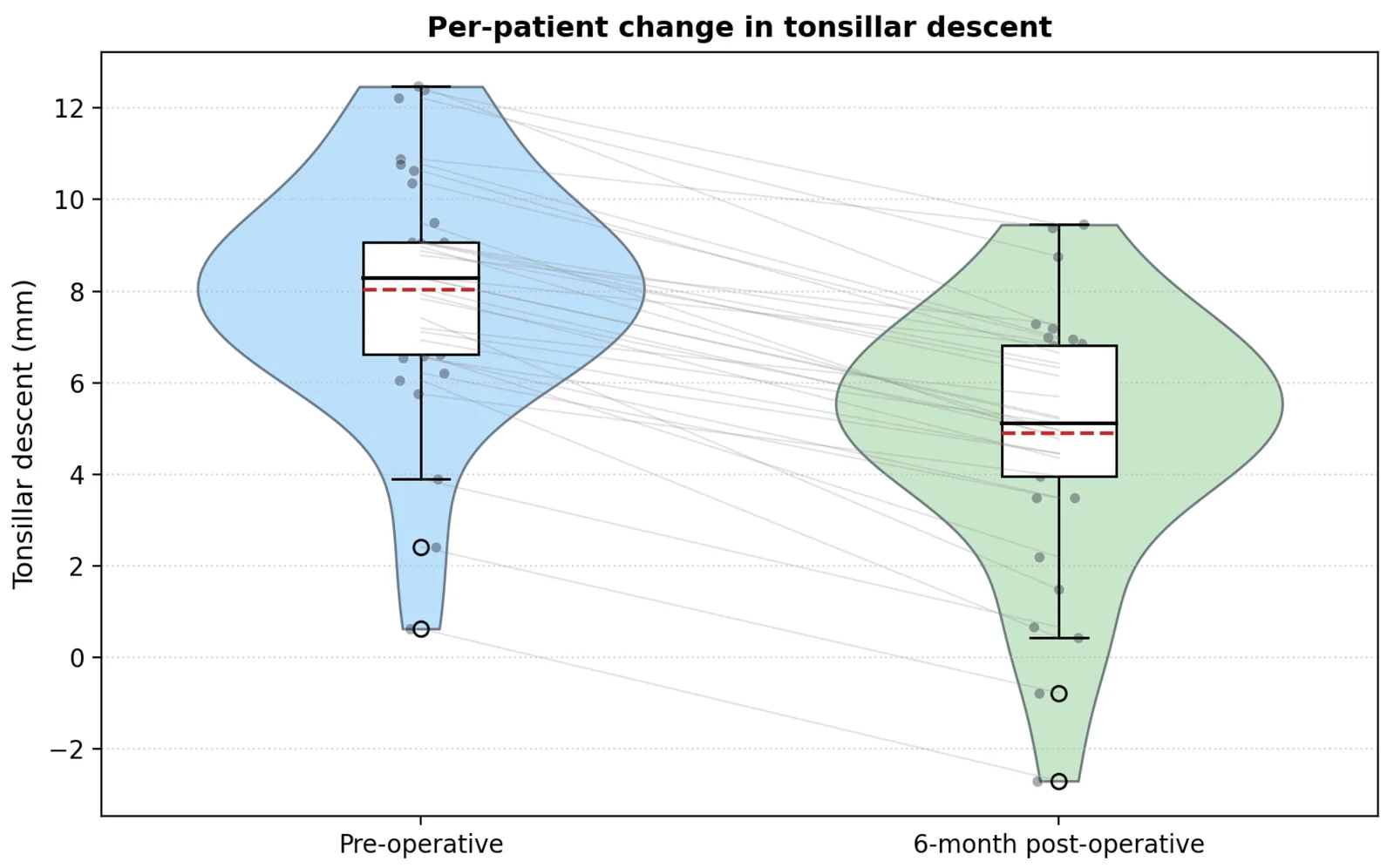
Per-patient change in tonsillar descent. In total, 29 of 33 patients (87.9%) achieved a tonsillar ascent of at least 2 mm at 6 months. Violins show the kernel density distribution of values (blue, pre-operative; green, 6-month post-operative); inner boxes show the median (black solid line) and IQR, with whiskers extending to 1.5 × IQR; red dashed lines, cohort mean; grey dots and thin grey lines, individual patients and their paired values; open circles, outliers. Negative values indicate a tonsillar tip above the McRae line.

Tonsillar descent decreased from 8.0 ± 2.8 mm to 4.9 ± 2.8 mm (Δ −3.1 mm, 95% CI −3.5 to −2.7; *p* < 0.001; d = −2.74, 95% CI −3.92 to −2.05), the largest effect size among the eight primary outcomes (Figure 3). Twenty-nine of 33 patients (87.9%) achieved a tonsillar ascent of at least 2 mm at 6 months, the pre-specified threshold for a clinically meaningful morphometric response (Section 2.7); four patients (12.1%) had ascent of less than 2 mm and are considered morphometric non-responders on the primary outcome. All four non-responders were female adults (mean age 41.5 years; mean pre-operative descent, 5.4 mm). None of the four non-responders required re-operation within the 6-month follow-up window.

**Figure 4:**
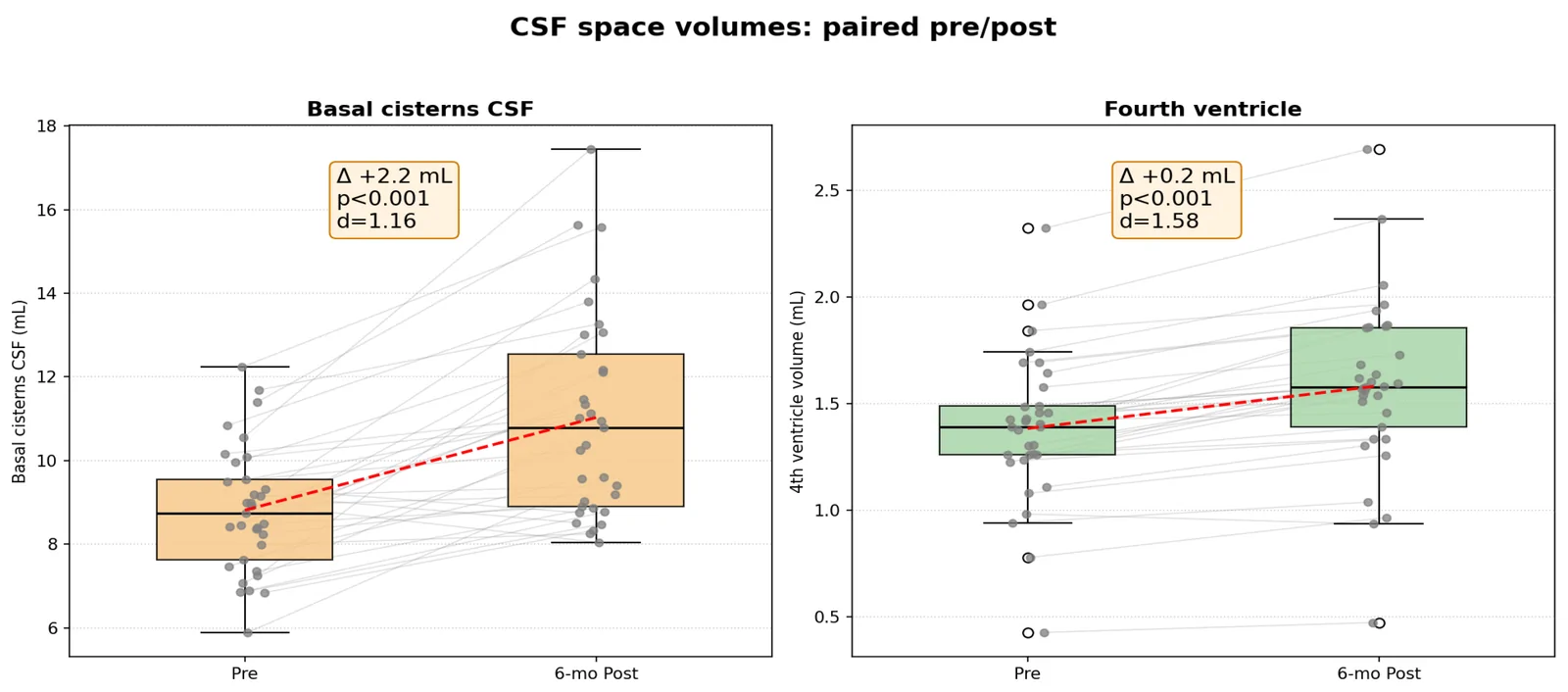
CSF space volumes: paired pre/post. Basal cisterns’ CSF and fourth ventricle volume both increased at 6 months (*p* < 0.001). Left, basal cisterns’ CSF volume (orange boxes); right, fourth ventricle volume (green boxes). Boxes show the median and interquartile range (IQR), and whiskers extend to 1.5 × IQR; grey dots, individual patients; thin grey lines, paired pre- and post-operative values of the same patient; open (white) circles, outliers beyond 1.5 × IQR; red dashed line, change in cohort mean.

Basal cisterns’ CSF volume increased from 8.8 ± 1.5 mL to 11.0 ± 2.4 mL (Δ + 2.2 mL, 95% CI 1.5–2.9; *p* < 0.001; d = 1.16, 95% CI 0.87–1.60), a relative increase of approximately 25%, which is the largest fractional change observed among the eight primary metrics (Figure 4).

Fourth ventricle volume increased from 1.4 ± 0.3 mL to 1.6 ± 0.4 mL (Δ + 0.2 mL, 95% CI 0.1–0.3; *p* < 0.001; d = 1.58, 95% CI 1.26–2.09), a modest absolute but proportionally larger (approximately 14%) relative increase.

Cerebellar volume (pre 115.6 ± 9.7 mL; post 117.6 ± 9.8 mL) increased by +2.0 mL (95% CI 1.2–2.9), a small but statistically significant change (paired t-test, *p* < 0.001; Cohen’s d = 0.86, 95% CI 0.50–1.53); brainstem volume (pre 22.9 ± 2.9 mL; post 22.8 ± 2.7 mL) was essentially unchanged (Δ −0.1 mL, 95% CI −0.4 to 0.2; Wilcoxon test, *p* = 0.22; d = −0.11, 95% CI −0.54 to 0.22).

**Figure 5:**
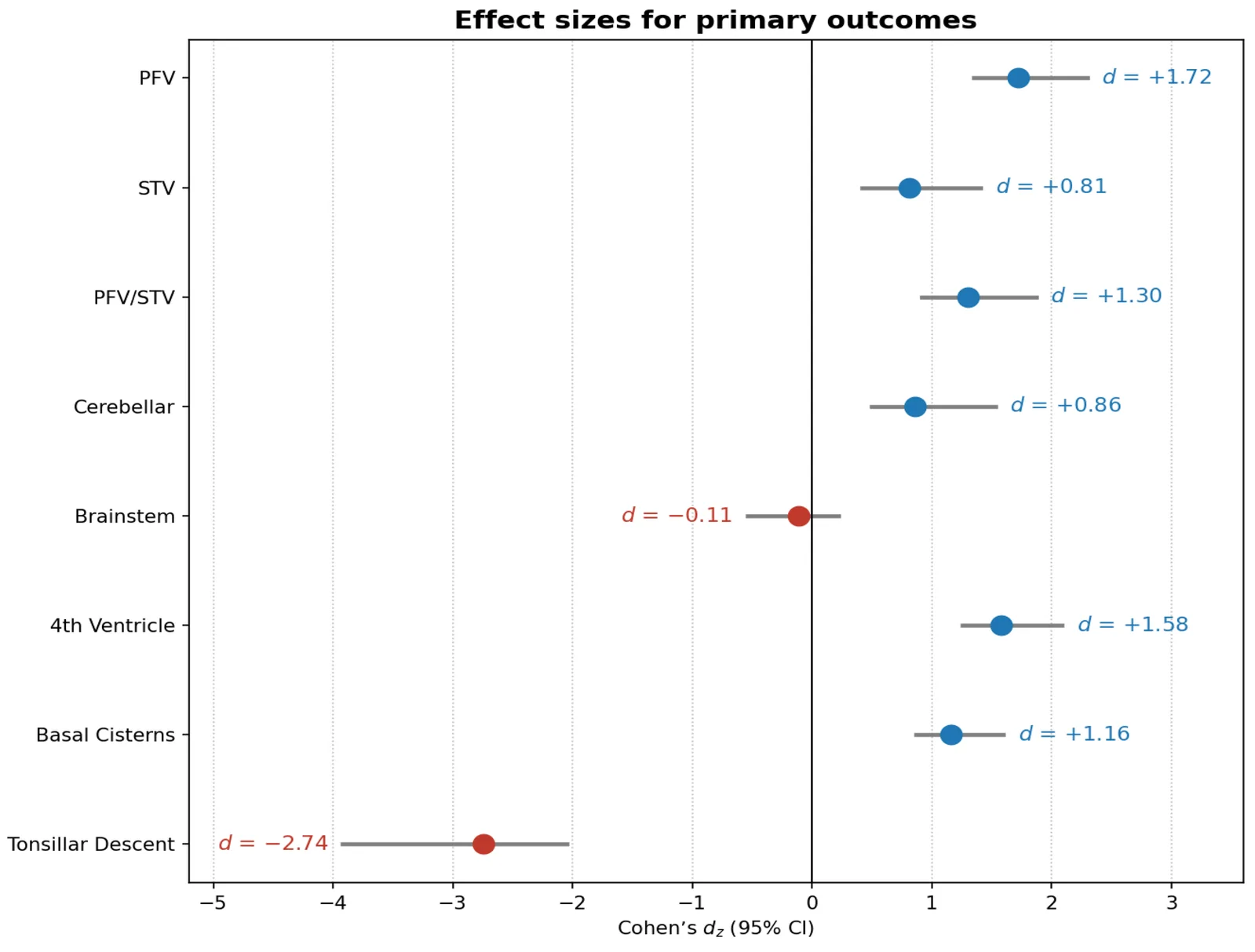
Effect sizes for primary outcomes (Cohen’s dz, 95% CI), analytic sample *N* = 33. Largest effect: Tonsillar descent (d = −2.74), followed by posterior fossa volume (d = 1.72). Blue markers, positive effect sizes (post-operative increase); red markers, negative effect sizes (post-operative decrease); grey horizontal bars, bootstrapped 95% CI; vertical black line, no effect.

After Benjamini–Hochberg correction across the eight primary metrics, seven outcomes remained significant at q < 0.05 (PFV, STV, PFV/STV, fourth ventricle, basal cisterns’ CSF, tonsillar descent, and cerebellar volume, all at q < 0.001), and one (brainstem volume, q = 0.22) did not (Figure 5).

### 3.3. Cerebellar Lobule Analysis

**Figure 6:**
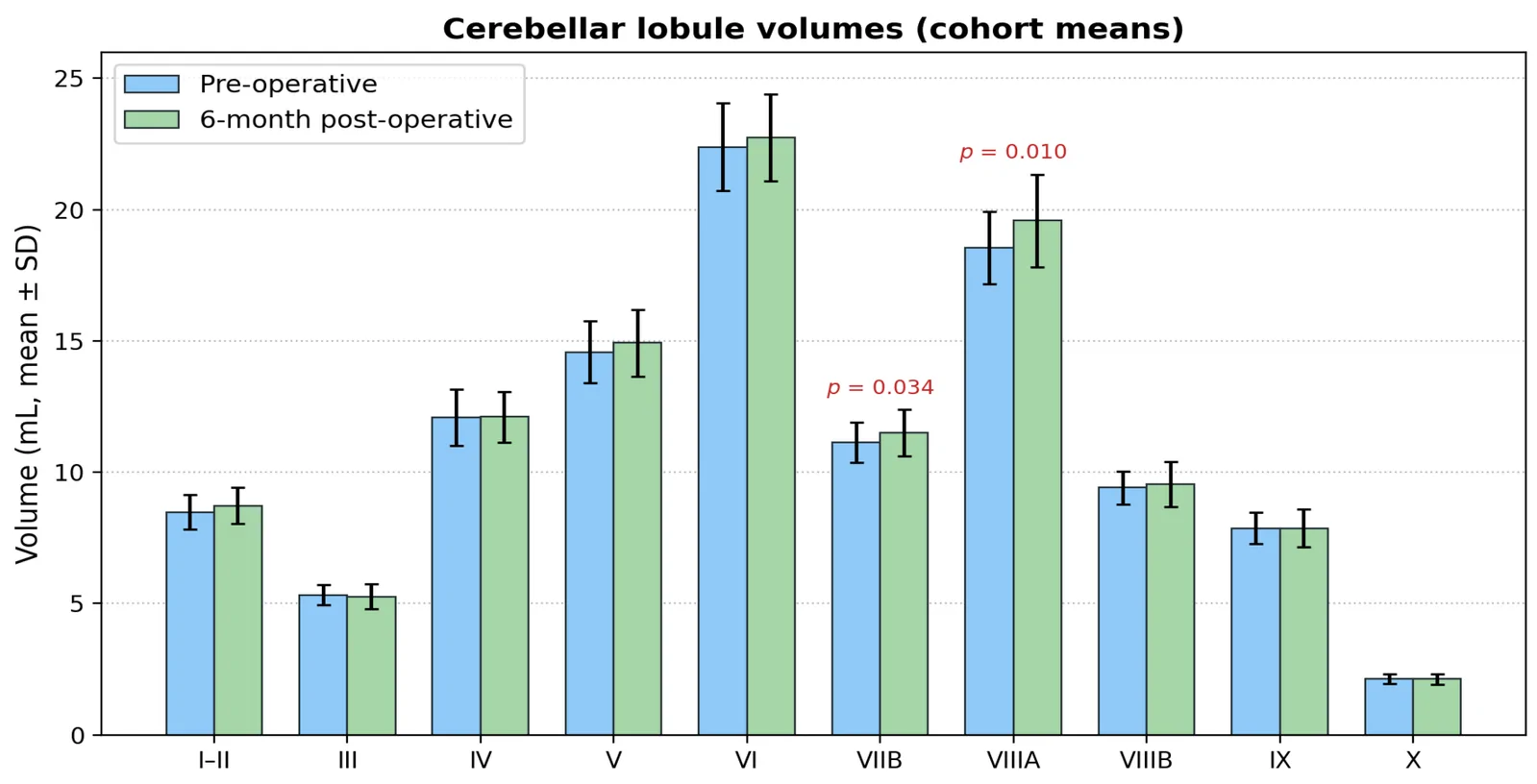
Cerebellar lobule volumes (cohort means). Ten cerebellar lobule regions (I–II, III, IV, V, VI, VIIB [Crus I + II], VIIIA, VIIIB, IX, X). Two regions (VIIB and VIIIA) reached uncorrected significance. Blue bars, pre-operative; green bars, 6-month post-operative; error bars, SD; red annotations, uncorrected Wilcoxon *p*-values.

Cerebellar lobule analysis (Table 3; Figure 6) showed directionally concordant, modest post-operative volume gains across nine of the ten lobule regions, with the flocculus (lobule X) unchanged. Two regions reached uncorrected statistical significance: VIIB (pre 11.1 ± 0.8 mL; post 11.5 ± 0.9 mL; *p* = 0.034) and VIIIA (pre 18.5 ± 1.4 mL; post 19.6 ± 1.8 mL; *p* = 0.010). The largest absolute change was observed in VIIIA (+1.04 mL), with additional gains in VI (+0.36 mL) and VIIB (+0.36 mL). After Benjamini–Hochberg correction across the ten lobule comparisons, neither result survived correction (q = 0.17 and q = 0.10, respectively), consistent with the modest sample size available for subregional analysis.

The anatomical pattern is informative. Lobules VIIB and VIIIA lie at the cerebellar convexity and the inferior semilunar lobule, bordering the lateral and dorsal cisterns; their post-operative expansion is consistent with re-expansion of cerebellar tissue into the decompressed cisterna magna and dorsal subarachnoid space. Lobule X (the flocculus) lies within the cerebellopontine angle and would not be expected to change with a suboccipital craniectomy. The relative stability of the more anterior lobules (I–II, III, IV, and V) is consistent with the anatomical reach of the decompression. Given that automated cerebellar lobule parcellation itself carries measurable inter-method variability even among current State-of-the-Art tools [14], and that the lobule-level analysis in this cohort would require substantially more patients to be adequately powered for FDR-corrected significance, these findings are best regarded as hypothesis-generating pending replication in a larger cohort. Whether longer follow-up (12–24 months) would reveal progressive lobule re-expansion is an open question and is one of the pre-specified analyses of a planned prospective extension.

### 3.4. Subgroup Analyses

Because the pediatric subgroup comprises only two patients, the exact Wilcoxon signed-rank test for this stratum can take only the values 1.000 or 0.500, regardless of effect magnitude, and is not a meaningful hypothesis test; the pediatric values in Table 4 are reported descriptively for transparency only and should not be used for inference. Subgroup analyses (Table 4) showed concordant directions of change across age and sex strata.

#### 3.4.1. Age

Posterior fossa volume delta in pediatric patients (*n* = 2) was +2.9 mL, and in adults (*n* = 31), it was +6.7 mL; the corresponding tonsillar ascent was −4.4 mm in pediatric patients, and −3.0 mm in adults. The pediatric estimates carry substantial uncertainty given the sample size of *n* = 2, and no formal interaction test was performed. The point estimates fall within 3.8 mL of each other, suggesting no large age-related heterogeneity in the structural response to PFD with superficial durotomy at 6 months, although the sample is far too small to draw a firm conclusion.

#### 3.4.2. Sex

Posterior fossa volume delta was +6.4 mL in females (*n* = 24) and +6.7 mL in males (*n* = 9); tonsillar ascent was −3.0 mm in females and −3.5 mm in males. As with age, no formal interaction test was performed. Baseline sex-related differences in posterior fossa morphology have been reported previously in Chiari I malformation [18], and the marginally more pronounced tonsillar ascent observed in males here is consistent with that broader literature, though this cohort is not powered to test for a sex-by-treatment interaction.

#### 3.4.3. Clinical Responder Analysis

Combining the morphometric and clinical responder criteria (tonsillar ascent of at least 2 mm AND self-reported improvement in the dominant symptom at 6 months), 26 of 33 patients (78.8%) met both. Of the seven patients who did not meet both criteria, four had an inadequate morphometric response, and three had adequate morphometric ascent but persistent myelopathic symptoms; the latter three are being followed for consideration of secondary duraplasty.

### 3.5. Adverse Events

No patient required re-operation within the 6-month follow-up window. Adverse events were infrequent and minor (Table 5). Four patients (12.1%) developed transient post-operative headache lasting a median of 4 days (range 2–9), all resolving with conservative management. One patient (3.0%) developed superficial wound dehiscence managed with local wound care and oral antibiotics. Two patients (6.1%) reported new-onset suboccipital numbness that resolved within 3 months. There were no cases of CSF leak, pseudomeningocele requiring intervention, aseptic meningitis, posterior fossa hematoma, or new neurological deficit.

## 4. Discussion

This study applied a fully automated, deep-learning segmentation pipeline to 1.5T 3D T1 imaging to detect and quantify structural change following PFD with superficial durotomy in Chiari I malformation. The observed pattern (posterior fossa expansion, basal cisterns’ CSF augmentation, fourth ventricle enlargement, and tonsillar ascent, with relative sparing of cerebellar and brainstem parenchymal volume) is consistent with the theoretical understanding of Chiari I pathophysiology and supports the feasibility of automated, reproducible, multi-structure morphometric assessment in routine Chiari I care.

### 4.1. Mechanistic Interpretation

The findings are consistent with a synthesis of the mesodermal and hydrodynamic theories of Chiari I pathophysiology [4,5,6,19]. Pre-operatively, a volumetrically constrained posterior fossa crowds its neural contents and obstructs CSF egress at the foramen magnum. After PFD with superficial durotomy, posterior fossa volume increased by approximately 3.9%, and the PFV/STV ratio rose by 0.4 percentage points, reflecting both the additional volume created by the bony decompression and re-accommodation of the cerebellum within the decompressed space. Because the posterior fossa mask used in this analysis includes the craniectomy defect itself, part of the measured PFV increase mechanically reflects the enlarged bony aperture created at surgery rather than biological tissue remodeling or improved CSF dynamics alone; this measurement characteristic should be considered when interpreting PFV as a marker of decompression adequacy, and metrics less directly confounded by the bony defect—basal cisterns’ CSF volume and tonsillar position—may better reflect the physiological response to decompression. The 25% relative increase in basal cisterns’ CSF volume is the most direct morphometric evidence in this dataset of restored extra-axial CSF space, consistent with the hydrodynamic model in which restoration of a low-resistance CSF outlet at the foramen magnum is reflected in augmented peri-cerebellar CSF volume. The proportionally smaller (approximately 14%) increase in fourth ventricle volume provides a corresponding, if more modest, signal of restored intra-axial CSF dynamics, and it should be interpreted cautiously given the small absolute magnitude of this structure. The 38.7% relative reduction in tonsillar descent is the most clinically interpretable single signal in this analysis and is consistent with the qualitative surgical experience that adequate bone decompression, including C1 laminectomy, produces visible tonsillar ascent in the large majority of patients.

### 4.2. Comparison with Prior Literature

Direct quantitative benchmarking of this automated multi-structure pipeline against prior series is limited by the scarcity of comparable, multi-structure, automated volumetric studies in the pre/post-operative Chiari I setting; to our knowledge, no prior report has combined nnU-Net-based posterior fossa/brainstem/ventricular segmentation with automated cerebellar lobule parcellation and automated tonsillar keypoint localization in this population. At the conceptual level, the direction and general magnitude of change observed here (modest posterior fossa volume expansion, more pronounced CSF-space augmentation, and preserved parenchymal volume) is consistent with the volumetric framework established by Trigylidas and colleagues in their pediatric series introducing the PFV/STV ratio as an outcome metric [11]. Automated cerebellar parcellation methods, including CERES, have themselves been shown in independent benchmarking to achieve good but imperfect agreement with manual delineation and with one another, which is a relevant caveat when interpreting lobule-level findings such as ours [13,14]. Because we are not aware of directly comparable published deltas for basal cisterns’ CSF volume or fourth ventricle volume obtained with an equivalent automated methodology, we present our findings as a candidate quantitative benchmark for future comparative work rather than as a replication of a specific prior numeric result. More broadly, these findings sit within a growing body of work applying quantitative three-dimensional methods to characterize postoperative structural change across surgical specialties, from cranial morphometry to spinal alignment assessed by three-dimensional posturography [17]; such methods share the advantage of capturing continuous, reproducible, multi-dimensional change, but they also share the limitation that, without a concurrent functional or symptomatic correlate, three-dimensional structural change alone cannot establish that the observed response translates into clinical benefit for an individual patient (Section 4.4).

### 4.3. Comparison with PFD with Duraplasty

Meta-analytic evidence comparing PFD with and without duraplasty consistently finds that adding duraplasty increases the risk of CSF-related complications, including CSF leak, pseudomeningocele, and aseptic meningitis, and lengthens operative time, while evidence for its incremental clinical benefit over bone-only decompression is more mixed, particularly in adult cohorts [9]. Superficial durotomy, as used in this study, was specifically designed as an intermediate strategy that divides only the outer dural layer while preserving the inner layer and arachnoid, intended to promote decompression-related re-expansion without opening the CSF space to the risks associated with full durotomy and duraplasty [10]. The 0% CSF leak rate, 0% pseudomeningocele rate, and 87.9% morphometric responder rate observed in this cohort are consistent with that intended risk–benefit profile, and echo the favorable complication and improvement rates reported in the original description of the technique [10]. The extent to which the smaller posterior fossa volume gain achieved with superficial durotomy relative to full duraplasty (which creates a larger surgical cavity through dural opening) translates into different long-term clinical outcomes cannot be determined from this study and would require a prospective comparative trial.

### 4.4. Clinical Implications

The morphometric evidence in this analysis carries several implications for surgical decision-making and patient counseling. First, an 87.9% morphometric response rate (tonsillar ascent of at least 2 mm) at 6 months after PFD with superficial durotomy provides a quantitative expectation that can be shared with patients during informed-consent discussions, which is more informative than a binary “surgery may help” framing. Second, the concordance of effect-size direction across age strata, while limited by the small pediatric subgroup in this cohort, is at least consistent with using PFD with superficial durotomy as a reasonable option across the age spectrum, including in pediatric patients in whom duraplasty carries a higher risk of CSF-related complications. Third, the 12.1% morphometric non-responder rate, together with published evidence that pre-operative imaging parameters do not reliably predict clinical surgical outcome [20], underscores that routine post-operative imaging and close clinical follow-up, rather than pre-operative imaging alone, are important for identifying patients whose decompression may be inadequate and who might be considered for secondary duraplasty. Fourth, the 25% fractional increase in basal cisterns’ CSF volume is the most direct morphometric signal of restored CSF dynamics identified in this series and may be a useful primary endpoint for future trials comparing surgical technique, given its large effect size and mechanistic interpretability. It should be emphasized, consistent with prior work showing that post-operative tonsillar position change does not reliably correlate with symptomatic outcome [20], that the morphometric responses reported here describe structural change and should not be equated with guaranteed clinical improvement; the concordance between morphometric and self-reported clinical responder status in this cohort (78.8%) illustrates that the two measures, while related, are not interchangeable.

### 4.5. Strengths

The strengths of this work include the following: (i) a fully automated, atlas-free segmentation pipeline that eliminates inter-rater variability and is well suited to multi-center deployment; (ii) multi-structure assessment spanning parenchymal, CSF, and osseous compartments from a single acquisition; (iii) cerebellar lobule-level resolution enabled by CERES, which is difficult to obtain with manual methods at this scale; (iv) a pre-specified statistical plan incorporating effect sizes, bootstrapped confidence intervals, and false-discovery-rate correction; (v) use of a 1.5T scanner platform that remains the clinical workhorse at many neuroscience centers, supporting generalizability; and (vi) a version-controlled, containerized pipeline intended to support independent reproduction.

### 4.6. Limitations

Several limitations should be considered. First, this is a single-center retrospective cohort from a tertiary practice, and the adult-predominant case mix (93.9% adult) limits inferences that can be drawn for pediatric Chiari I malformation specifically. Second, the cohort size of 33 limits the power of subgroup analyses and precludes formal interaction testing; a planned prospective extension targets a substantially larger cohort. Third, the follow-up window is 6 months, and longer-term morphometric trajectory (12–24 months) is unknown. Fourth, the protocol did not include phase-contrast CSF flow quantification, which would provide a complementary dynamic assessment of CSF dynamics. Fifth, the segmentation pipeline was trained primarily on 1.5T Siemens Aera data and may require re-training or domain adaptation for other vendors or field strengths. Sixth, the McRae line is the most widely used reference for tonsillar descent but not the only candidate; alternative landmarks such as the obex-to-tonsil distance may provide complementary information, and published evidence indicates that post-operative tonsillar position change correlates imperfectly with clinical symptomatic outcome, a caveat that should temper over-interpretation of the morphometric responder rate reported here [20]. Seventh, the cerebellar lobule-level analysis is underpowered for the small effect sizes observed and should be regarded as exploratory. Eighth, contrary to the original pre-specified plan, the 11 patients with pre-operative syrinx were retained in the primary volumetric analysis rather than excluded, a deviation adopted to preserve statistical power across strata (Section 3.1); syrinx status may be an unmeasured source of heterogeneity in the pooled effect sizes, and a dedicated syrinx-stratified analysis is planned for the prospective extension of this cohort. Ninth, external quantitative benchmarking against other automated multi-structure volumetric series was constrained by the limited number of directly comparable published studies using an equivalent automated methodology in this specific pre/post-operative population, as discussed in Section 4.2. Tenth, the cohort was anatomically heterogeneous with respect to tonsillar descent severity, syrinx morphology, and prior craniocervical instrumentation (two patients with previous C1–C2 posterior fusion); this heterogeneity was not incorporated into the statistical model, and the pooled effect sizes should be interpreted as an average across a clinically and radiologically diverse population rather than as characterizing a single homogeneous phenotype. Eleventh, segmentation reliability (Section 2.6) was benchmarked against manually corrected masks in only 3 of 33 patients (a 10% subset), and the lowest agreement was observed for the basal cisterns (median Dice 0.79)—one of the primary outcomes with the largest effect size in this analysis; this is a modest basis for reliability estimation across four distinct segmentation tools, and although no gross segmentation failures were identified on visual review of flagged outlier cases (Section 2.4), undetected structure-specific segmentation error contributing to the magnitude of the basal cisterns and fourth ventricle effect sizes cannot be excluded; expanding the manually validated subset to at least 10 patients is planned for the prospective extension of this cohort.

### 4.7. Future Directions

This analysis supports several specific future directions: prospective multi-center validation of the pipeline across vendor platforms at 1.5T; extension of the protocol to 3T to assess whether higher signal-to-noise ratio improves lobule-level resolution; integration of phase-contrast CSF flow metrics to relate structural and dynamic change; development of an outcome-prediction model incorporating pre-operative morphometry and clinical variables; extension of the segmentation pipeline to the craniocervical junction CSF spaces and spinal cord for patients with syrinx; and development of a clinician-facing tool that generates an automated pre/post-operative morphometry report from a routine 3D T1 acquisition to support real-time surgical decision-making.

## 5. Conclusions

Automated volumetric analysis of 1.5T 3D T1 imaging demonstrated significant posterior fossa expansion, basal cisterns’ CSF augmentation, fourth ventricle enlargement, and tonsillar ascent following PFD with superficial durotomy in a 33-patient cohort. The pipeline, integrating nnU-Net, CERES, TotalSegmentator, and nnDetection, delivers reproducible, multi-structure morphometry suitable for both retrospective cohort analysis and prospective surgical outcome studies.

The reported effect sizes (PFV + 6.5 mL, d = 1.72; basal cisterns’ CSF + 2.2 mL, d = 1.16; and tonsillar descent −3.1 mm, d = −2.74) provide a quantitative benchmark for sample-size estimation in future trials comparing surgical technique (PFD alone, PFD with superficial durotomy, and PFD with duraplasty) and for counseling patients on expected structural change at 6 months. The 87.9% morphometric responder rate establishes a benchmark for bone-only/superficial-durotomy decompression, while the 12.1% non-responder rate underscores the need for close clinical and radiological follow-up and further work to identify pre-operative predictors of an inadequate response.

## Figures and Tables

**Table 1:** Demographic and baseline characteristics.

Characteristic	Value
*N*	33
Female, *n* (%)	24 (72.7)
Male, *n* (%)	9 (27.3)
Pediatric (<18 y), *n* (%)	2 (6.1)
Adult (≥18 y), *n* (%)	31 (93.9)
Age, years (mean ± SD)	39.2 ± 13.5
Age, years (median [IQR])	39.0 [32.0–44.0]
Symptom duration, months (mean ± SD)	38.2 ± 41.6

**Table 2:** Primary volumetric outcomes, pre- vs. 6-month post-operative.

Metric	Preop (Mean ± SD)	Postop (Mean ± SD)	Delta (Mean ± SD)	Test	*p*-Value	*p* (FDR-BH)	Cohen’s d
PFV (mL)	166.0 ± 12.9	172.5 ± 13.1	6.5 ± 3.8	paired t	<0.001	<0.001	1.72
STV (mL)	1063.4 ± 105.2	1070.1 ± 106.6	6.7 ± 8.3	paired t	<0.001	<0.001	0.81
PFV/STV (%)	15.5 ± 1.9	16.0 ± 1.9	0.4 ± 0.3	paired t	<0.001	<0.001	1.30
Cerebellar (mL)	115.6 ± 9.7	117.6 ± 9.8	2.0 ± 2.4	paired t	<0.001	<0.001	0.86
Brainstem (mL)	22.9 ± 2.9	22.8 ± 2.7	−0.1 ± 0.9	Wilcoxon	0.2208	0.2208	−0.11
4th Ventricle (mL)	1.4 ± 0.3	1.6 ± 0.4	0.2 ± 0.1	paired t	<0.001	<0.001	1.58
Basal cisterns’ CSF (mL)	8.8 ± 1.5	11.0 ± 2.4	2.2 ± 1.9	paired t	<0.001	<0.001	1.16
Tonsillar descent (mm)	8.0 ± 2.8	4.9 ± 2.8	−3.1 ± 1.1	paired t	<0.001	<0.001	−2.74

Note: All statistics in this table (*N* = 33, the full analytic sample; Section 3.1) use Cohen’s d reported as dz for paired samples (mean within-subject difference ÷ SD of the within-subject differences), with 95% CI from 10,000 bootstrap resamples of the paired differences (Section 2.7).

**Table 3:** Cerebellar lobule volumes, pre- vs. post-operative.

Lobule	Preop (mL, Mean ± SD)	Postop (mL, Mean ± SD)	Delta (mL)	*p* (Wilcoxon)
I–II	8.5 ± 0.7	8.7 ± 0.7	+0.23	0.13
III	5.3 ± 0.4	5.3 ± 0.5	−0.06	0.55
IV	12.1 ± 1.1	12.1 ± 1.0	+0.03	0.97
V	14.6 ± 1.2	14.9 ± 1.3	+0.35	0.20
VI	22.4 ± 1.7	22.8 ± 1.7	+0.36	0.51
VIIB (Crus I + II)	11.1 ± 0.8	11.5 ± 0.9	+0.36	0.034
VIIIA	18.5 ± 1.4	19.6 ± 1.8	+1.04	0.010
VIIIB	9.4 ± 0.6	9.5 ± 0.9	+0.13	0.42
IX	7.9 ± 0.6	7.9 ± 0.7	0.00	0.96
X (flocculus)	2.1 ± 0.2	2.1 ± 0.2	0.00	0.97

**Table 4:** Subgroup analyses by age and sex.

Stratifier	Metric	Delta	*p*
Pediatric (*n* = 2)	PFV (mL)	+2.9	1.000
Pediatric (*n* = 2)	Tonsillar descent (mm)	−4.4	0.500
Adult (*n* = 31)	PFV (mL)	+6.7	<0.001
Adult (*n* = 31)	Tonsillar descent (mm)	−3.0	<0.001
Female (*n* = 24)	PFV (mL)	+6.4	<0.001
Female (*n* = 24)	Tonsillar descent (mm)	−3.0	<0.001
Male (*n* = 9)	PFV (mL)	+6.7	0.012
Male (*n* = 9)	Tonsillar descent (mm)	−3.5	0.004

**Table 5:** Adverse events at 6-month follow-up.

Event	*n* (%)	Management	Outcome
Transient headache	4 (12.1)	Conservative	Resolved
Superficial wound dehiscence	1 (3.0)	Conservative	Resolved
Suboccipital numbness (transient)	2 (6.1)	Observation	Resolved < 3 mo
CSF leak	0 (0.0)	-	-
Pseudomeningocele requiring intervention	0 (0.0)	-	-
Aseptic meningitis	0 (0.0)	-	-
Posterior fossa hematoma	0 (0.0)	-	-
New neurological deficit	0 (0.0)	-	-
Re-operation (within 6 mo)	0 (0.0)	-	-

## Data Availability

The data presented in this study are available upon reasonable request from the corresponding author. The data are not publicly available due to patient privacy restrictions on the underlying clinical imaging dataset.
